# High-Performance Polymer Blends: Manufacturing of Polyetherimide (PEI)–Polycarbonate (PC)-Based Filaments for 3D Printing

**DOI:** 10.3390/polym16233384

**Published:** 2024-11-30

**Authors:** Shikha Singh, Pascal Hubert

**Affiliations:** 1 Department of Mechanical Engineering, McGill University, Montreal, QC H3A 0C3, Canada; pascal.hubert@mcgill.ca; 2 CREPEC—Research Centre for High-Performance Polymer and Composite Systems, Montreal, QC H3A 0C3, Canada

**Keywords:** high-temperature thermoplastic blends, additive manufacturing, 3D printing, high-temperature polymers, mechanical properties

## Abstract

The demand for high-performance polymers in 3D printing continues to grow due to their ability to produce intricate and complex structures. However, commercially available high-temperature 3D printing materials often exhibit limitations such as brittleness, warping, thermal sensitivity, and high costs, highlighting the need for advanced filament development. This study investigates the fabrication of polyetherimide (PEI) and polycarbonate (PC) blends via melt extrusion to enhance material properties for stable additive manufacturing. The addition of PC improved the processability of the blends, enabling successful extrusion at temperatures ranging from 290 to 310 °C. Differential scanning calorimetry (DSC) confirmed a shift in the softening temperature (T) of PEI, indicating effective blending. To further improve the properties of the PEI:PC blends, 1 wt% of a compatibilizer was incorporated, resulting in homogeneous microstructures as observed through scanning electron microscopy (SEM). The optimized PEI:PC (70:30) blend with compatibilizer (1 wt%) demonstrated a 49% higher storage modulus than neat PEI and a 40% greater storage modulus than ULTEM9085. Moreover, reduced melt viscosity facilitated consistent and stable printing, making these materials highly suitable for applications in aerospace and transportation, where performance and reliability are critical.

## 1. Introduction

Additive manufacturing (AM), commonly referred to as three-dimensional (3D) printing, has garnered significant attention in both academia and industry due to its versatility and wide range of applications. These include sectors such as automotive [1], aerospace [2,3], healthcare [4,5,6], construction [7], electronics [8], and sports [9]. According to ISO/ASTM 52900:2021 standards [10], AM techniques are categorized into seven primary types: (1) material extrusion (ME), (2) material jetting (MJ), (3) binder jetting (BJ), (4) sheet lamination (SL), (5) vat photopolymerization (VP), (6) powder bed fusion (PBF), and (7) directed energy deposition (DED) [11,12]. Each technique offers unique advantages in terms of speed, resolution, and cost, providing users with a variety of options depending on application requirements.

Within the material extrusion (MEX) category, fused filament fabrication (FFF), also known commercially as fused deposition modeling (FDM), is among the most widely adopted techniques [13,14]. MEX involves selectively depositing a thermoplastic polymer through a heated nozzle in a layer-by-layer fashion to construct free-standing structures. The FDM process operates by heating the filament to its molten state, extruding it through a nozzle, and forming layers with dimensional accuracy on the order of 100 µm [15]. Key parameters that influence the quality of printed parts include nozzle temperature, bed temperature, print speed, layer height, and infill density. These factors collectively determine adhesion, precision, and mechanical properties. Compared to other AM techniques such as PBF and VP, MEX offers several advantages, including scalability to print small and large parts, cost-efficiency, and the ability to fabricate complex geometries. These features have made MEX particularly valuable in industries like automotive and aerospace for prototyping, tooling, and manufacturing lightweight components [11,16].

**Figure 1:**
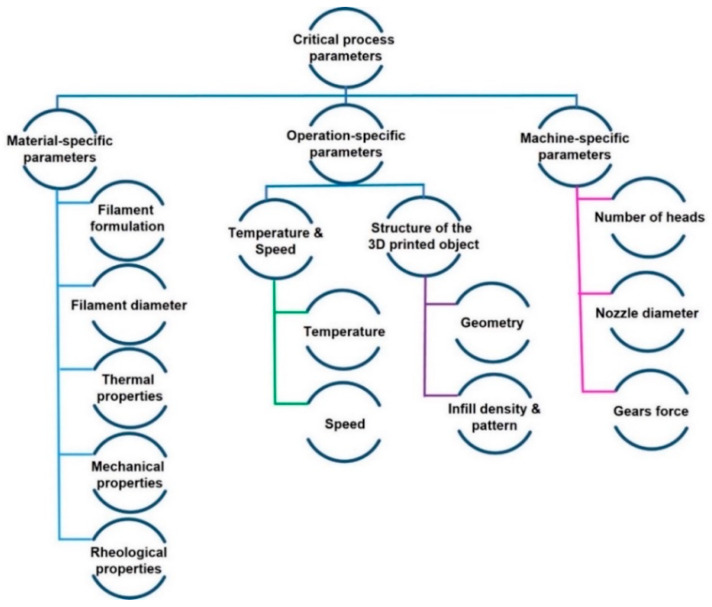
Critical process parameters used in Fused Filament Fabrication (FFF) inspired by [21].

Hot-melt extrusion (HME) is a widely utilized technique in Material Extrusion (MEX)-based 3D printing for processing thermoplastic polymers [17]. Thermoplastics, such as polyetherimide (PEI), polylactic acid (PLA), and acrylonitrile butadiene styrene (ABS), are characterized by their ability to soften and flow upon heating and solidify upon cooling. This reversible thermal behavior makes them ideal for extrusion-based processes, where the material is heated to its processing temperature, extruded through a nozzle, and deposited layer-by-layer to build the desired geometry [18]. HME not only enables precise control over material deposition but also facilitates the incorporation of additives and fillers to tailor the properties of the printed parts, making it a versatile approach for producing functional components in various applications [19]. Despite its advantages, the transition of MEX from a prototyping method to a full-scale manufacturing solution faces several challenges [20]. These challenges can be broadly categorized into three groups: material-specific, operation-specific, and machine-specific [21] (Figure 1). Addressing these limitations is essential for advancing the capabilities of MEX as a reliable manufacturing technology.

Material-specific parameters are related to the physicochemical properties of the filament, such as rheological, thermal, and mechanical properties. Operation-specific parameters encompass the printing processing conditions, such as printing temperature, printing speed, and infill density, which influence the final quality of the printed parts. Machine-specific parameters consist of the exact characteristics of the FFF printer and its parts. FFF exhibits poor surface quality, which is determined by nozzle dimensions and polymer viscoelasticity [22,23], low build speed [9], and limited material options compared to traditional processing methods [24]. Most importantly, MEX has crucial limitations for practical applications owing to weak and anisotropic mechanical properties originating from poor diffusion and entanglement of chains between filaments during the deposition process, resulting in a weak interlayer bond formation [25]. Herein, the focus is given to tackling material- and operation-specific challenges.

Commercial filaments of high-performance polymers, available on the market for MEX, are limited due to their inherent properties, such as high melt viscosity, inherent crystallinity, and moisture absorption. The main challenges in manufacturing novel materials are associated with poor thermal stability and mechanical properties, buckling due to high pressure, and slippage between the drive wheels. Furthermore, the extrusion process must also be optimized for high-performance polymeric materials. To mitigate these problems, filaments require specific rheological properties such as viscosity, thermal, and mechanical properties [26]. The cost of current commercial filaments used in MEX, especially high-performance polymeric filaments, is still very high, around USD 200–600 per kilogram. Therefore, it has become crucial to develop novel materials that can be efficient in terms of performance and cost-effectiveness. In this context, polymer blending has been recognized as the most versatile and economical method due to its ability to produce materials that can satisfy complex demands for performance. This work used polyether imide (PEI) and polycarbonate (PC) polymers to prepare the polymer blends. PEI is an amorphous thermoplastic polymer with ether groups that provide high melt processability. PEI has a high glass transition temperature of around 217 °C, which helps the material maintain structural integrity at elevated temperatures and supports high thermo-oxidative stability. PC is also an amorphous polymer with very high toughness and resistance. PEI was selected for its good mechanical properties and chemical resistance, and PC was chosen due to its better impact and toughness properties [27,28]. Many polymeric blends have been developed for decades for different applications. However, very few articles have been published on high-performance polymers, especially for MEX [3,29,30]. Liu et al. [31] prepared PC/poly(butylene adipate-coterephthalate) (PBAT) blends by adding methyl methacrylate–butadiene–styrene terpolymer (MBS) to overcome the toughness issues. Blanco et al. [32] developed a polymer blend with PEI and polyethylene terephthalate glycol (PETG) for MEX. They investigated the thermal properties of the blends to improve the processability of the pristine PEI. It was observed that adding PEG lowered the glass transition and thus the viscosity, which means it improved the processability. Magri et al. [33] manufactured a blend of PEI with polyether ether ketone (PEEK) for MEX. Adding PEI into PEEK improved the service temperature by 20 °C and decreased the processing temperature by 20 °C without compromising the tensile strength. Wang et al. [34] prepared a sandwich structure of PEEK: PEI using MEX and thoroughly studied the mechanical properties of the printed part. Lewis and co-workers also studied the PEEK: PEI blend [35]. High-performance polymer blends used for MEX are focused on PEI: PEEK combinations. To the best of our knowledge, only a couple of research articles (2–3) have been published so far on the development of polyetherimide (PEI) and polycarbonate (PC) blends for MEX. For instance, Blanco et al. [36] manufactured PEI: PC blends with different compositions and studied their thermal properties of the prepared blends. Cicala et al. [37] prepared PEI: PC blends and thoroughly investigated the rheological and thermo-mechanical properties of the prepared blends. In both cases, only a minimal amount (45 g) of PEI/PC polymer blends were manufactured by batch mixing. A commercially available blend of PEI: PC filaments from Stratasys, named ULTEM9085, is still expensive, and the patent is not publicly available. Furthermore, there is scope to improve the properties of ULTEM9085 for better printability. To compare the properties of the PEI: PC blends with commercial filaments, it is essential to first develop the materials on a large scale using an industrial-scale twin extruder in filament form. Transferring the lab-tested materials to an industrial scale further needs process optimizations, especially when manufacturing materials with new compositions.

The main objective of this study was to develop a high-temperature PEI: PC filament using a twin extrusion technique for larger-scale production and compare the properties of the developed filaments to commercial ULTEM9085 filament. The work was fragmented into three segments: (i) filament development, (ii) print optimization, and (iii) performance evaluation. It has always been challenging to blend PEI and PC because of the differences in the nature of the polymers and their properties, which result in poor blend properties. To mitigate poor adhesion between PEI: PC, a compatibilizer was added to the blends, and it was expected that the compatibilizer would not only enhance the performance of the filament but also improve the printability due to better interfacial adhesion between PEI and PC phase. The extrusion process optimization was carried out to develop the filaments with correct ovality and diameter. The print optimization was carried out by employing the design of the experiment (DOE). Furthermore, the mechanical properties of all the developed filaments were tested to confirm that they can be used in 3D printing. The rheological, thermal, and structural properties of the filaments were characterized before their use in printing. Finally, the developed filament used for 3D printing and processing parameters such as print speed, extrusion temperature, print bed temperature, and chamber temperature were optimized for a stable printing.

## 2. Materials and Methods

### 2.1. Materials

An amorphous, popularly used industrial grade polyetherimide (PEI) known as ULTEM1000 was purchased from SABIC, Canada. Polycarbonate (PC) (LUPOY GN1006FL CLEAR) was supplied by Chase Plastics, Canada. To improve surface morphology and facilitate the processability of the PEI: PC blends, a compatibilizer (provided by Polyscope, Geleen, The Netherlands) was added to the blend. The melt flow index (MFI), density, and average molecular weights of PEI and PC are given as follows: 9 g/10 min, 22 g/10 min, 1.27 g/cm^3^, 1.2 g/cm^3^, 20,000 to 60,000 g/mol, and 30,000 to 40,000 g/mol, respectively. Moreover, compatibilizer was expected to enhance the printability, thermal, and mechanical properties. All the materials, including PEI, PC, and compatibilizer, were in pellet form. Finally, ULTEM9085 filaments were purchased from Stratasys and the properties of the developed filaments of PEI: PC with and without compatibilizer were compared.

### 2.2. Methods

Filaments of PEI: PC blends were prepared from raw materials using twin extrusion in pellet form, and then the developed polymeric filaments were characterized by different techniques to gain insight into their thermal, rheological, mechanical, and structural properties. Finally, the filaments were then used to print samples, followed by characterization.

### 2.3. Filament Fabrication

All materials were thoroughly dried at 120 °C overnight to eliminate absorbed moisture and prevent degradation during processing. The fabrication of filament was carried out in two sequential steps: (1) materials blending and (2) filament spooling. Initially, the dried polymer pellets were mixed in predetermined proportions and introduced into the hopper of a twin-screw extruder (Coperion, Stuttgart, Germany). The materials were uniformly blended, melted, and extruded through a die with a 2.5 mm diameter and a length-to-diameter (L/D) ratio of 30:1, forming a continuous filament. The extruded filament was subsequently wound onto a spool for further use. The extruder consists of 11 heating zones, and the zone temperature can be defined as (Ti), which was precisely set to T1:250 °C, T2:260 °C, T3:270 °C, T4:280 °C, T5:285 °C, T6:295 °C, T7:295 °C, T8:310 °C, T9:300 °C, T10:300 °C, and T11; 295 °C, with a die temperature maintained at 295 °C. The extrusion process was conducted at a screw speed of 100 rpm, with a material feed rate of 1.0 kg/h. The filament diameter was regulated by adjusting the winding speed and subsequently collected on an empty spool. The diameter and ovality measurements were periodically taken during the spool winding process. Measurements of filaments were carried out on an electronic system using a laser micrometer (Laserlinc Triton 312, Fairborn, OH, USA) along the entire length of the spool, and the average and standard deviation (STD) were calculated. The average diameter and ovality of the produced filaments were 1.74–1.79 mm and 0.059–0.065 mm, respectively. The filament spool was stored in a dry cabinet (Dr. Storage X2B) below 5% RH, and the moisture content of the filament was measured by a moisture analyzer on Brookfield Ametek Computrac Vapor PRO XL (Middleboro, MA, USA); as per the ASTM D7191 [38], to measure the relative moisture content of filament samples, the samples were cut from the spool. Drying the filaments before printing was crucial as it could affect the print quality. Therefore, the moisture content of all the developed filaments was measured and it was below 0.04%. The developed materials were designated according to the ratio of the polymer and compatibilizer used, as outlined in Table 1. 

### 2.4. Three-Dimensional Printing of Materials

The prepared PEI blend filaments were printed using an AON3D printer (M2-2020, AON3D, Montreal, QC, Canada), known for its capability to handle high-temperature polymers. Printing high-performance polymers like PEI presents significant challenges due to their high melt viscosity and sensitivity to process conditions. A Taguchi Design of Experiments (DoE) approach was used to systematically evaluate the effects of processing parameters on the storage modulus of the developed filament (see Table 2). The objective was to optimize printing conditions to achieve the maximum storage modulus. Three process parameters (factors) were studied, each at three levels: (i) **Nozzle Temperature**: 340 °C, 350 °C, 360 °C; (ii) **Bed Temperature**: 180 °C, 190 °C, 200 °C; (iii) **Chamber Temperature**: 100 °C, 120 °C, 130 °C. Taguchi L9 orthogonal array was chosen to study the three factors at three levels with nine experimental runs using Minitab 20 software. Signal-to-Noise (S/N) ratios were calculated using the “larger-is-better” criterion to analyze the effect of each factor on the storage modulus. Factor-level averages of S/N ratios were computed to identify optimal levels. The predicted S/N ratio and corresponding storage modulus for the optimal parameter’s combinations were calculated.

After optimizing these parameters, all the DMA samples were printed with an infill density of 100%, a nozzle diameter of 0.4 mm, a raster angle of ±45, and a layer thickness of 0.2 mm. The print speed was 30 to 50 mm/s.

### 2.5. Characterizations

#### 2.5.1. Thermal Study

The thermal degradation of neat PEI and PEI: PC filaments was analyzed using thermos-gravimetric analysis (TGA) on a TA-Q500 machine with 10 °C/min ramp rate from room temperature to 900 °C under air and nitrogen. The parameters associated with the physical properties of the PEI: PC blends, such as temperature at 5% and 30% mass loss, (T5%) and (T30%), respectively, were evaluated and compared with ULTEM9085. Char yield, which is the difference between the mass of a sample heated in inert gas and air divided by the original sample weight, was determined for all the formulations. Differential scanning calorimetry (DSC) was used to evaluate the thermal properties of PEI: PC blends. DSC was performed on TA DSC-Q100 with a ramp of 10 °C/min. Furthermore, the effect of compatibilizer on the thermal properties of the blends was also evaluated by DSC. Polymer–polymer interaction plays an important role in the polymer blends; therefore, the interaction parameter (χ) was also calculated using DSC by employing the Flory–Huggins equation reported in the literature [39].

#### 2.5.2. Rheological Analysis

Rheological properties play a key role in determining the printing properties of polymeric materials. In this study, the rheological measurements were carried out on an Anton Paar (MCR302) rheometer equipped with parallel plates of 25 mm diameter. Produced filaments were cut into pieces and charged on pre-heated lower plate and then the upper part of the plate was closed to reach a gap of 1.0 mm. Before the test, an amplitude sweep was performed to find the linear viscoelastic region (LVR). The melting of samples occurred, and excess materials were scraped with a steel spatula. The recommended printing temperature for PEI is 350 °C. Therefore, the rheological analysis was carried out at this temperature. However, PC was also added to the PEI blends that have lower melting temperatures than PEI. Thus, rheological characterization was also performed at different temperatures to optimize the filament fabrication and printing process. The frequency sweep test was conducted at 310 °C, 330 °C, and 350 °C, and the frequency varied from 0.1 to 100 rad/s.

#### 2.5.3. Mechanical Testing

Mechanical properties of all the developed filaments were tested based on ASTMD638 [40] (Type I sample) using a Capstone grip with a diameter of 96.4 mm equipped with a load cell 5 kN on a tensile testing machine (MTS, Insight 5 kN). The test was performed at a crosshead speed of 200 mm/min. Five samples per condition were tested, and the average and standard deviation were calculated. The diameter of the filament was measured at three different points, and the average was used to calculate it. Thermo-mechanical properties are crucial for understanding the flowability and printability of materials. The viscoelastic behavior of the blends was investigated using a dynamic mechanical analyzer (DMA) (TA-Q800) instrument in a three-point bending mode. Rectangular shape samples were printed with a dimension of 12.5 mm × 60.0 mm × 2.5 mm. Tests were performed at a rate of 3 °C/min with a frequency of 1 Hz. The temperature was ramped from 30 to 200 °C. A total of three replicates were tested, and an average was taken. Glass transition temperature (Tg) was determined from the loss modulus (G″) peak.

#### 2.5.4. Morphological Analysis

Optical microscopy (OM) and scanning electron microscopy (SEM) were used to examine the surface of PEI: PC polymeric filaments. A Nikon L150 (Tokyo, Japan) reflective optical microscope was used to observe the developed filament surfaces both in parallel and perpendicular to the length of the filament. The fractured surface of the filaments was used to analyze the texture of surfaces on a Hitachi SU 3500 Variable Pressure Electron Microscope (VP-SEM). The surface of cryo-fractured filaments was coated with a thin layer of gold (15–20 nm) to obtain a better image and avoid the charging effects. Cryo-fracture samples were analyzed on an SU8230 with a voltage of 3 kV.

#### 2.5.5. Fourier Transform Infrared (FTIR) Analysis

FTIR analysis was performed with a BRUKER, Billerica, MA, USA (Vertex 70) ATR spectrometer. The filaments of all materials, including neat PEI, PEI: PC (70:30), PEI: PC (70:30) X1, and ULTEM9085, were cut into pellet form and analyzed in the wavenumber range of 500 to 3500 cm^−1^ with a resolution of 4 cm^−1^.

## 3. Results

### 3.1. Thermal Properties

**Figure 2:**
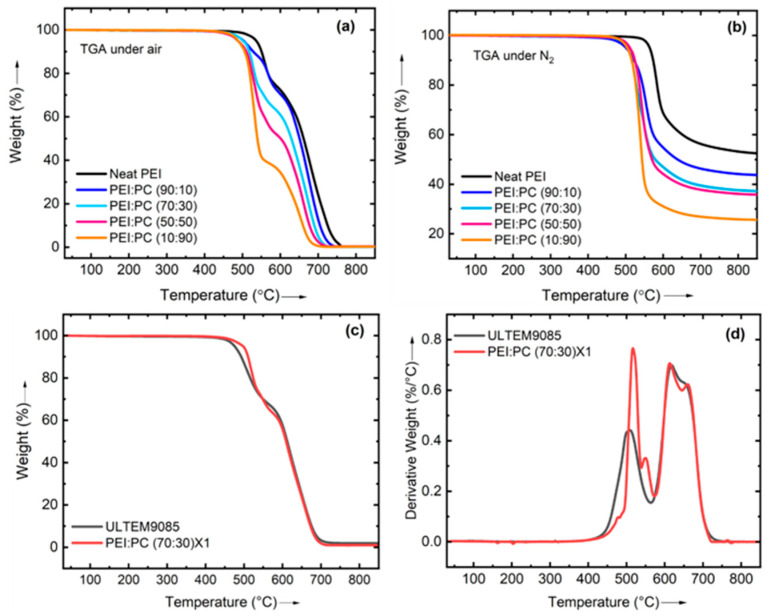
TGA curves of PEI and its blends under (**a**) air and (**b**) nitrogen at 10 °C/min and comparison of thermal stability of ULTEM9085 with PEI: PC 70:30X1 after 3D printing showing (**c**) TGA curves and (**d**) DTG curves.

Thermal properties play a vital role in the development and performance of new filaments for 3D printing. For instance, they dictate the (i) printability and extrusion temperature, (ii) layer adhesion and strength, (iii) warpage and shrinkage, (iv) heat resistance and stability, and (v) surface finish. Furthermore, filaments with tailored properties are essential to meet regulatory requirements and functional needs. Hence, it is crucial to investigate the thermal properties of the newly developed filaments to expand the range of applications and improve the overall quality and reliability of 3D-printed parts. In the present study, the thermal degradation behavior of the blends was evaluated under an air and nitrogen environment to understand the thermal stability of manufactured filaments and avoid the degradation of materials during 3D printing at elevated temperatures. TGA also helps find printing parameters such as nozzle temperature, heated bed temperature, and printing speed. Filaments with lower thermal decomposition temperatures require lower printing temperatures to prevent thermal degradation and maintain print quality. Filaments’ temperature versus percentage weight loss curves are presented in Figure 2. All the filaments were thermally stable above 400 °C under air and nitrogen. Neat PEI had the highest thermal stability, and when the PC content increased, the thermal stability decreased because of the lower thermal stability of the PC. Among the PEI: PC blends, PEI: PC (70:30) showed good thermal stability. Furthermore, the addition of compatibilizer and its effect on the thermal properties of PEI: PC (70:30) X1 blends were examined, and the results are compiled in Table 3. It can be seen from Table 1 that adding a compatibilizer enhanced the thermal stability of the PEI: PC (70:30) X1, resulting in a 22 °C higher thermal degradation temperature (Td5%) under air compared to PEI: PC (70:30). Figure 2c,d shows comparative TGA and DTG curves of ULTEM9085 and PEI: PC (70:30) X1. It is visible from Figure 2c that the thermal stability of PEI: PC (70:30) X1 (red line) was higher than the ULTEM9085 (black line). In the DTG graph, two peaks were observed in the materials: ULTEM9085 and PEI: PC (70:30) X1. The intensity of peak one (in red) was higher than ULTEM9085. However, no significant difference in peak two was observed. FTIR was performed and is described in a later section to understand further peak shifts.

It is worth mentioning that PEI: PC (70:30) X1 exhibited the highest char yield among different formulations of PEI: PC blends (see Table S1). The char yield of PEI: PC (70:30) X1 increased to 716% and 790% with respect to neat PEI and PEI: PC (70:30). This may be because of the better interface between the PEI: PC phase developed due to the compatibilizer. It has been reported in the literature that a higher char yield means that the materials will be able to maintain their mass at elevated temperatures [41]. In the current study, TGA results indicate that adding the compatibilizer not only increased the char yield under air but also reduced the mass loss rate. It can be seen from Table S1 that char residues under an inert atmosphere reduced compared to the neat PEI. Comparing PEI: PC (70:30) to PEI: PC (70:30) X1, the char yield was increased from 0.12% to 0.98%. Char residues under inert conditions are related to the flame-retardant capabilities of the samples by the limiting oxygen index (LOI) that can be estimated using the Van Krevelen equation [42]. LOI can be calculated as follows: LOI = 17.5 + 0.4 × CR, where CR is the char residue function under nitrogen at 900 °C in percentage. A material is called flammable when its LOI is ≤26% [42]. In the present study, the LOI values of all materials were higher than 26%, which indicates that the developed materials are not flammable.

The newly developed filaments must have a melting temperature or flow temperature compatible with commercial printers [43]. The melting point of many commercial filaments is too high. That is why most materials, despite being developed as filaments, cannot be printed by MEX 3D printing [43]. In this work, both the polymers, PEI and PC, are amorphous; therefore, their thermal behavior, especially glass transition temperatures of neat and PEI: PC blends, were evaluated using DSC (Table 2). DSC thermographs of the developed filaments are shown in Figure S1. Two distinct glass transition temperatures for all the composition of PEI: PC blends were observed: one at 212 °C corresponding to PEI-rich phase and the other at 145 °C corresponding to PC-rich phase. Similar results were noticed by Blanco et al. [32]. The presence of two Tg peaks indicates that PEI: PC blends are immiscible or partially miscible blends. By adding a compatibilizer to the PEI: PC (70:30) blend, the glass transition of the PC phase was changed. This shift in the glass transition temperature of polymer blends was due to the blends’ softening or melting temperature.

Polymer components can soften at widely different temperatures; however, compatibility is determined by miscibility parameters, which describe the interaction of polymer repeat units. In this work, the Flory–Huggins interaction parameter (χ) of the polymer blend was used to calculate and understand the miscibility between the polymer phases. It is well known that heterogeneous blends of polymer exhibit two glass transitions. Partial miscibility results in a shift in the degree of component mixing. According to Kim and Burn, the apparent weight fraction of polymer components in each phase of partially miscible blends can be calculated by reformulating the Fox equation, as mentioned below.

(1) $$\frac{1}{T_{g}}=\frac{W_{1}}{T_{g1}}+\frac{W_{2}}{T_{g2}}$$

where $W_{1}$ and $W_{2}$ are the weight fraction of the polymer components of the blend and $T_{g1}$ and $T_{g2}$ are the glass transition temperatures of polymer 1 and polymer 2, respectively.

Equation (1) may be rearranged to calculate the apparent weight fractions of the polymers:

(2) $$W_{1}^{\prime }=\frac{T_{g1},b\left(T_{g1}-T_{g2}\right)}{T_{g1},b\left(T_{g1}-T_{g2}\right)}$$

(3) $$W_{2}^{\prime \prime }=\frac{T_{g1},b\left(T_{g2b\;}-T_{g1}\right)}{T_{g2},b\left(T_{g2}-T_{g1}\right)}$$

where $W_{1}^{\prime }$ and $W_{2}^{\prime \prime }$ are the apparent weight fraction of polymer 1 and 2 and $T_{g1b\;}$ and $T_{g2b\;}$ are the observed $T_{g\;}$ of polymer 1 and 2 in the blend $T_{g1}$ and $T_{g2}$ are the $T_{g}$ of polymer 1 and 2.

Apparent volume fractions (ø) calculated from the apparent weight fractions, and eventually, chi (χ) parameters calculated using the Kim and Burns equations.

(4) $$\chi_{12}=\frac{\ln\left(\frac{\phi_{1}^{\prime \prime }}{\phi_{1}^{\prime }}\right)+\left(1-m_{\frac{1}{}}m_{2}\right)\left(\phi_{2}^{\prime \prime }-\phi_{2}^{\prime }\right)}{m_{1}\left(\phi_{2}^{\prime 2}-\phi_{2}^{\prime \prime 2}\;\right)}$$

(5) $$\chi_{21}=\frac{\ln\left(\frac{\phi_{2}^{\prime \prime }}{\phi_{2}^{\prime }}\right)+\left(1-m_{\frac{2}{}}m_{1}\right)\left(\phi_{1}^{\prime \prime }-\phi_{1}^{\prime }\right)}{m_{1}\left(\phi_{1}^{\prime 2}-\phi_{1}^{\prime \prime 2}\;\right)}$$

where $\phi_{1}$ and $\phi_{2}$ are the volume fraction of polymer 1 and 2, respectively. Two conjugate phases are doted by single and double primes and m is essentially the degree of polymerization and relates to the ratio between molar volume of one polymer and an arbitrary fictitious volume. Equations (4) and (5) should give the same calculated value; however, it may vary depending on the experimental error. The magnitude of the $\chi_{12}$ depends on molecular parameters, for instance, molar volumes. The $\chi_{12}$ for PEI: PC blends was in the range of 0.235–0.768, as reported by Chun et al. [44]. This indicates that the blends have good miscibility in PEI and PC phases. The miscibility between the polymer and other materials in the blend, such as compatibilizer, can significantly affect the system’s rheological properties, eventually impacting the processability, properties, and performance of printed parts.

### 3.2. Rheological Properties

Rheology plays an important role in determining a polymer’s printability; hence, the rheological properties, such as the complex viscosity (η*), storage modulus (G′), and loss modulus (G″) of the PEI and their blends with PC, were evaluated. According to Duty et al. [45], a material is printable if it fulfills the following four criteria: (i) the material must be extrudable through a given diameter of nozzle at a specific flow rate; (ii) extruded material must be able to hold the desired shape; (iii) extruded structure must be able to bridge a specific gap and serve as a mechanically sound substrate; and (iv) the finally deposited structure must retain geometrical stability while being cooled at room temperature. Rheological properties directly control all the criteria mentioned above, and therefore, tailoring the polymer feedstock by the addition of other polymers and/or compatibilizer to ensure appropriate rheological performance is essential for achieving the printed part’s stability and performance. The most important parameter that determines the printability of a polymeric material is melt viscosity. The main reason for blending PEI with PC is to reduce the high melt viscosity of PEI and improve the processability. Usually, the shear rate in liquefier is very high. Adequate shear thinning is required as shear thinning influences the ability of the melt to be forced through the nozzle, and it also helps acquire the required shape and structure after deposition. The volume flow rate (Q) can be calculated using the radius of nozzle exit.

(6) $$Q=\pi r^{2}v$$

The corresponding apparent shear rate of the PEI: PC blends at the nozzle wall can be semi-empirically determined from Equation (2), as given below:

(7) $${\dot{\gamma }}_{app}=\frac{4Q_{v}}{\pi r^{3}}$$

where $\dot{\gamma }$ is the printing speed, r is the radius of the nozzle, and Q is the volumetric flow that depends on the extrusion parameters and the polymers used.

In this study, a printing speed of 30 mm/s to 50 mm/s and a printer nozzle radius (r) of 0.4 mm equates to a flow rate of 15.0 mm^3^/s and 25.1 mm^3^/s, respectively, and consequently, an apparent shear rate ${(\overset{˙˙}{\gamma }}_{app})$ of 298.5 s^−1^ and 500 s^−1^ was obtained. It can be concluded from the results that the developed materials have good melt viscosity needed for printing.

**Figure 3:**
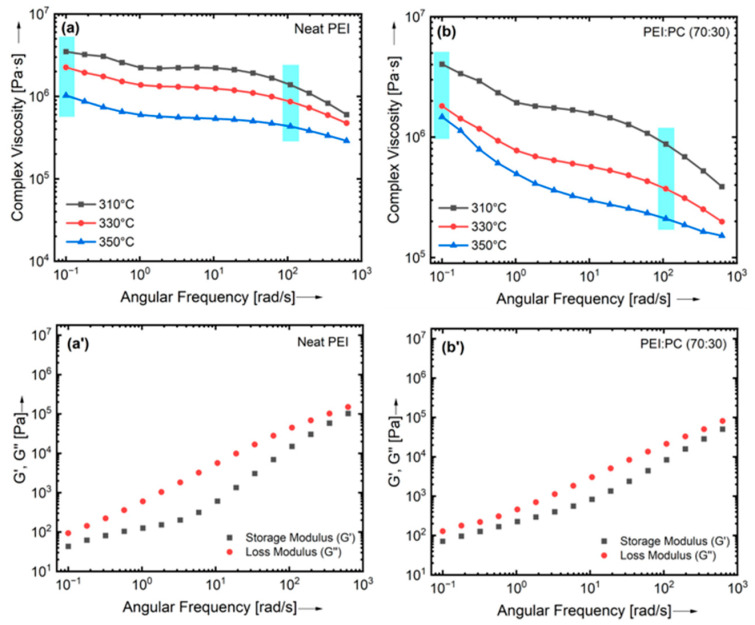
Rheological curves of (**a**,**a′**) neat PEI and (**b**,**b′**) PEI: PC (70:30) showing the complex viscosity, storage, and loss modulus curves. The blue line in the graphs represents the change in shear-thing behavior while changing the temperature.

The complex viscosities versus frequency curves and the storage (G′) and loss modulus (G″) results with respect to angular frequency for neat PEI and PEI: PC (70:30) blends at three different isothermal temperatures (310 °C, 330 °C, and 350 °C) are shown in Figure 3. The complex viscosities of both neat PEI and PEI: PC (70:30) decreased with increasing the temperature from 310 °C to 350 °C. The addition of PC into PEI slightly influenced the viscosity of the PEI: PC (70:30) blends due to shear thinning behavior and resulted in an increase in complex viscosity at all temperatures and frequencies.

**Figure 4:**
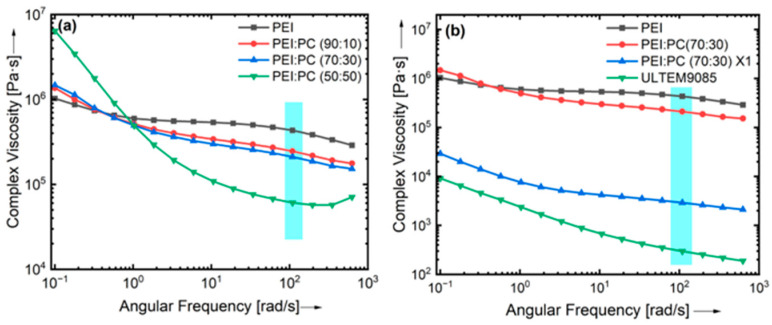
Complex viscosity versus angular frequency curves of (**a**) neat PEI and PEI: PC blends and (**b**) PEI, PEI: PC (70:30), and PEI: PC (70:30) X1 at isothermal 350 °C and their comparison with ULTEM9085. The blue line in the graphs represents the range of complex viscosity for printable materials.

Furthermore, the complex viscosities of PEI: PC blends with different compositions were determined and compared with compatibilizer and ULTEM9085. The rheological curves are presented in Figure 4. It can be seen from Figure 4a that the complex viscosities of PEI: PC blends were overlapped at a frequency of 1 (rad/s) and decreased up to 100 (rad/s). PEI: PC (50:50) showed a drastic change in complex viscosity. This could be because of the poor compatibility of the PEI and PC phases. Figure 4b shows the complex viscosities of neat PEI and PEI: PC (70:30), and their comparison with PEI: PC (70:30) X1 and ULTEM9085. The addition of the compatibilizer decreased the complex viscosity of the PEI: PC (70:30) X1 blends. However, it was higher than that of ULTEM9085. Cicala et al. [37] reported a decrease in complex viscosity of ULTEM9085 compared to the neat PEI at 350 °C over the entire frequency range. This decrease in the complex viscosity of the ULTEM9085 could be due to the better molecular interaction between the polymer phases. It has been reported in the literature that the ideal viscosity range for MEX materials is on the order of 102–105 Pa.s [46]. The results obtained in this work are in accord with trends, which further confirms that the PEI: PC with compatibilizer can improve printability.

### 3.3. Mechanical Properties

**Figure 5:**
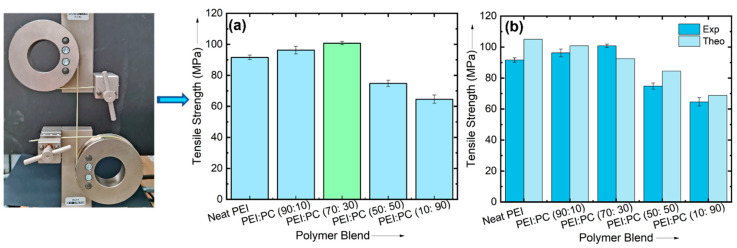
Mechanical properties of PEI: PC blends obtained from tensile test: (**a**) comparative mechanical properties of different polymer blend compositions, and (**b**) comparison between experimental and theoretical tensile strength of polymer blends.

Before using the developed filaments for printing, it is essential to test the mechanical properties of the materials to understand if the filaments can bear the load while passing through the gears during 3D printing. Figure 5 shows results of tensile testing for all PEI: PC filaments’ compositions. Neat PEI exhibited a tensile strength of 92 MPa. Among all compositions of PEI: PC blends, the lowest tensile strength was shown by PEI: PC (90:10) due to higher content of the PC phase into the PEI: PC blend. It can be seen from the bar graph that the highest mechanical properties were observed in the PEI: PC (70:30) blend. The enhanced mechanical properties of PEI: PC (70:30) could be because of favorable changes in the morphology of the PEI and PC phase. The experimental results were finally compared with the theoretical ones, and similar trends were observed, as shown in Figure 5 b. PEI: PC (70:30) showed the highest mechanical properties. Therefore, this composition was chosen for 3D printing, and further characterization was carried out to understand the reasons for the better mechanical properties of PEI: PC (70:30) blends.

### 3.4. Three-Dimensional Printing Optimization

In the realm of 3D printing, where process variations can have a profound impact on the performance of printed parts, DOE is a crucial tool for minimizing process variability and enhancing product reliability, efficiency, and quality. The Taguchi method was applied to analyze the impact of process parameters such as nozzle temperature, bed temperature, and chamber temperature on the storage modulus of PEIPC 70:30 filaments. The mean Signal-to-Noise (S/N) ratios for each factor at their respective levels were calculated to identify optimal settings. The following observations were made:

1. **Nozzle Temperature**: The mean S/N ratio decreased as the nozzle temperature increased from 340 °C to 360 °C. The highest mean S/N ratio (6.01) was observed at 340 °C, indicating this is the optimal level for maximizing storage modulus.
2. **Bed Temperature**: The S/N ratio exhibited a parabolic trend, with the highest mean S/N ratio (5.62) at 190 °C. This indicates that 190 °C is likely to be the optimal bed temperature.
3. **Chamber Temperature**: The mean S/N ratio peaked at 120 °C (5.56), followed by a decline at 130 °C. Therefore, 120 °C is the optimal chamber temperature.

Mean Signal-to-Noise (S/N) ratios indicated the optimal settings: 340 °C for nozzle temperature, 190 °C for bed temperature, and 120 °C for chamber temperature. These conditions, as illustrated in Figure S3, were used for printing all DMA samples, ensuring maximum performance.

### 3.5. Thermo-Mechanical Properties

**Figure 6:**
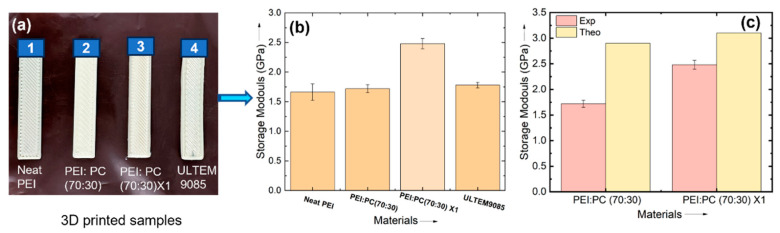
(**a**) DMA printed samples and (**b**) comparison of storage modulus of neat PEI, PEI: PC (70:30), PEI: PC (70:30) X1, and ULTEM9085; (**c**) comparative graph of experimental versus theoretical storage modulus of PEI: PC (70:30) and PEI: PC (70:30) X1.

To assess the impact of the compatibilizer on the thermo-mechanical properties of the blends, the storage moduli of neat PEI, PEI: PC (70:30), PEI: PC (70:30) X1, and ULTEM9085 were systematically compared. As illustrated in Figure 6, the PEI: PC (70:30) X1 blend achieved the highest storage modulus of 2.48 GPa, representing a 49% enhancement over neat PEI and a 40% improvement relative to the commercially available ULTEM9085 filament. This substantial improvement is attributed to enhanced interfacial compatibility between the PEI and PC phases, facilitated by the addition of the compatibilizer. Furthermore, the experimental data closely corresponded with theoretical predictions (Figure 6b), with minor deviations attributed to the exclusion of printing parameters in the theoretical model. Notably, the storage modulus observed in this study (2.48 GPa) surpasses previous results reported by Cicala et al. [37], where PEI:PC blends with polyethylene terephthalate glycol-modified (PETG) exhibited significantly lower values (0.3 GPa with 30 wt% PC and 5–10 wt% PETG). Unlike those results, which showed a decline in modulus relative to neat PEI, our study demonstrates that incorporating up to 30 wt% PC not only maintains but exceeds the modulus of ULTEM9085. These findings highlight the critical role of compatibilizers in optimizing the mechanical properties of PEI-based blends for advanced applications.

### 3.6. Morphological Properties

**Figure 7:**
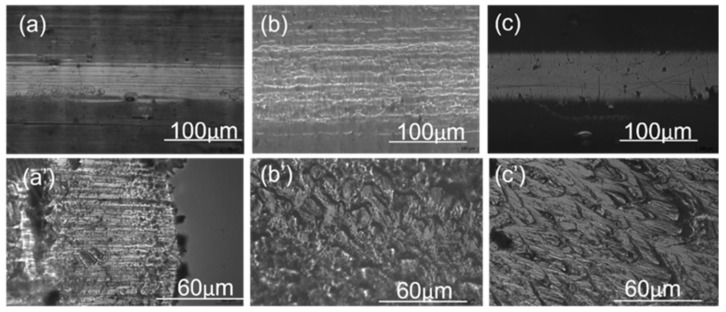
Optical microscopic images (**a**–**c**); surface and cross-section (**a′**–**c′**) of PEI, PEI: PC (70:30), and PC filaments.

To investigate the structure–property relationships, filaments were analyzed using optical microscopy (OM). Figure 7 presents optical micrographs of neat PEI, PC, and PEI: PC (70:30) blends. Discontinuous structures were evident in neat PEI (Figure 7a,a′), whereas PEI: PC (70:30) blends exhibited oriented fibril-like structures (Figure 7b,b′). In contrast, neat PC displayed more oriented structures, which is attributed to its high ductility (Figure 7c,c′). To further confirm these observations, scanning electron microscopy (SEM) was performed.

**Figure 8:**
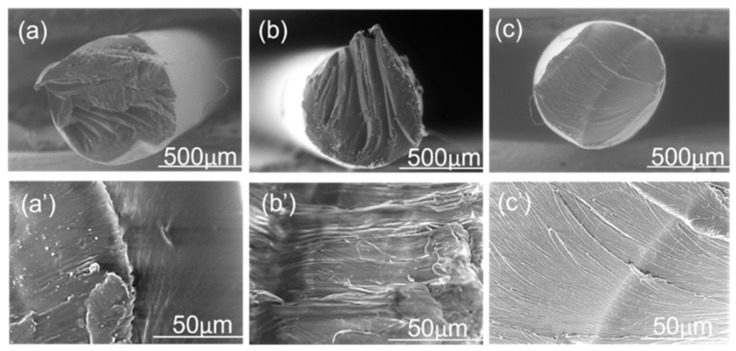
Scanning microscopic images of cross-section of neat (**a**,**a′**) PEI, (**b**,**b′**) PEI: PC (70:30), and (**c**,**c′**) PC filaments.

Figure 8 presents the SEM micrographs of neat PEI, PEI: PC (70:30) blends, and neat PC. The morphology of neat PEI (Figure 8a,a′) is in accordance with the observations from optical micrographs, showing discontinuous structures. In the PEI: PC (70:30) blends (Figure 8b,b′), the surface exhibits distinct fiber-like structures. Neat PC (Figure 8c,c′) reveals highly oriented fibrillar structures, which is consistent with its inherently ductile nature. The SEM analysis aligns well with the optical microscopy findings, providing complementary evidence of the observed morphologies. Blanco et al. [36] also observed co-continuous morphology in PEI: PC blends.

**Figure 9:**
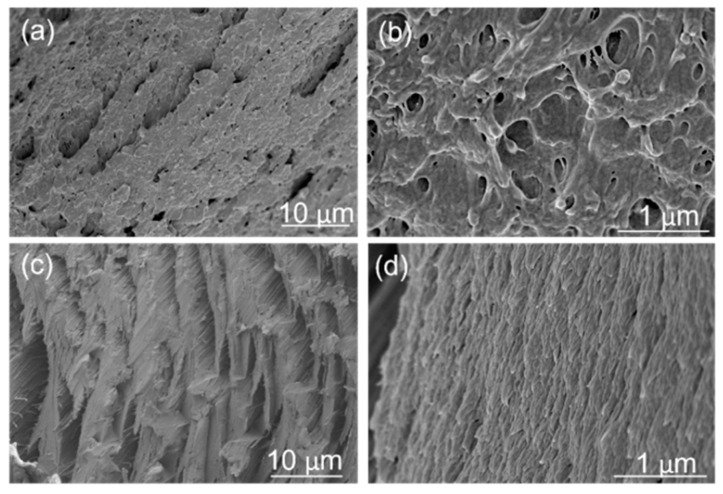
Scanning microscopic images of cryogenically fractured surfaces of (**a**,**b**) PEI: PC (70:30) and (**c**,**d**) PEI: PC (70:30) X1.

Figure 9a,b compares the cryogenically fractured SEM micrographs for PEI: PC (70:30) with and without compatibilizer. PEI: PC (70:30) without compatibilizer exhibited a droplet-like morphology. However, after adding the compatibilizer, the morphology changes to a more fibril-like structure, as shown in Figure 9c,d. The morphology of two-phase components can be determined by many factors, such as the processing method (shear rates, etc.), the melt viscosity ratio, and the interfacial tension between the dispersed phase and the continuous phase [47]. Here, the differences in morphology of the PEI: PC (70:30) and PEI: PC (70:30) X1 blends are due to their different melt viscosities. When 1 wt% of compatibilizer was added to the PEI: PC (70:30), it displayed a finer morphology. This improvement in PEI: PC (70:30) X1 was attributed to improved microstructural homogeneity at higher extrusion temperatures. It is worth mentioning that no fiber was pulled out from the PEI matrix during fracture, indicating strong adhesion between the compatibilizer and the polymer. This further indicates the role of compatibilizer in the PEI: PC (70:30) blend and reveals why PEI: PC (70:30) and PEI: PC (70:30) X1 exhibited better mechanical performance compared to neat PEI and ULTEM9085.

### 3.7. Strucutral Properties

**Figure 10:**
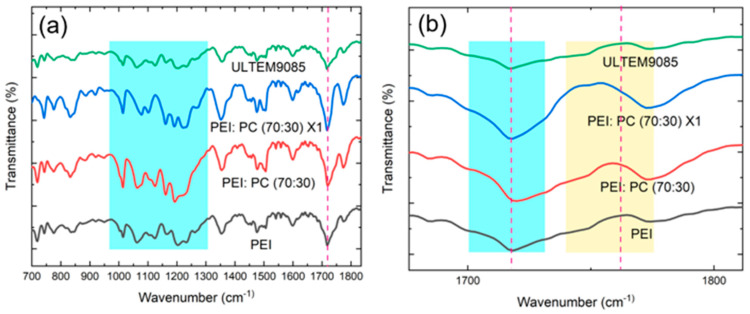
(**a**) FTIR spectrum of the neat PEI, PEI: PC (70:30), PEI: PC (70:30) X1, and ULTEM9085; (**b**) the effect of blending and compatibilizer on the C=O stretching bands in PEI. The blue and yellow zones represent C=O stretching associated with the imide ring of PEI at 1718 cm^−1^ (in-phase) and 1780 cm^−1^ (out-phase), respectively, showing how the C=O peak is shifting in the blends.

The ATR-FTIR spectra of neat PEI, PEI: PC (70:30), PEI: PC (70:30) X1, and ULTEM9085 samples were recorded (see Figure 10) to understand the role of compatibilizer and polymer–polymer interactions in PEI: PC blends. The characteristic peaks of PEI can be seen in all the materials. The peak at 910 cm^−1^ is attributed to the out-of-plane deformation of -NH_2_, the peak at 1045 cm^−1^ correlates with the stretching vibration of C-N bond, and the peak at 1600 cm^−1^ is attributed to the deformation of N-H bond. The spectrum of neat PEI exhibits a typical imide group peak at 1780 cm^−1^ (C=O out-of-phase stretching) and 1718 cm^−1^ (C=O in-phase stretching) corresponding to the ketone asymmetrical stretching vibration and carbonyl symmetrical vibration, respectively [33,48]. Figure 10b displays the C=O stretching bands of neat PEI and PEI in PC blends with compatibilizer and their comparison with ULTEM9085. The polymer mixing and compatibilizer highly influence the frequency of the C=O out-of-phase stretching. The frequency of this band increases slightly by 2 cm^−1^ from neat PEI to the PEI: PC (70:30) blend. The intensity of the carbonyl peak expands and shifts in the case of PEI: PC (70:30) and becomes broader in PEI: PC (70:30) X1. These intensity variations have been attributed to the effect of blending on the coplanarity of the two imide rings bridged by the phenylene group. Changing the coplanarity of these two imide rings can alter the intensity of C=O out-of-phase stretching, but it cannot affect the intensity of C=O in-phase stretching [49]. The observed effect of blending on the C=O out-of-phase stretching of PEI implies that some of the groups in PC interact favorably with the C=O groups of PEI. The change in the conformation of the entire imide ring causes this change in intensity [49]. Two leading bands are associated with the vibrations of the imide rings in PEI: 1380 cm^−1^ and 1357 cm^−1^. Like the C=O in-phase stretching, the frequency of band 1380 cm^−1^ is not affected by the blending. However, blending strongly affects the frequency of band 1357 cm^−1^. The sharp peak situated at 1590 cm^−1^ corresponds to the C-C stretching of the aromatic ring, and the peaks at 1230 and 1174 cm^−1^ are attributed to C-O-C stretching of the aromatic ether [33]. As expected for PEI: PC (70:30) X1, the FTIR spectrum exhibited all characteristic bands of both PEI and PC polymers. Based on these observations, it is reasonable to infer that blending and addition of compatibilizers impacted the interactions of the PEI and PC.

**Figure 11:**
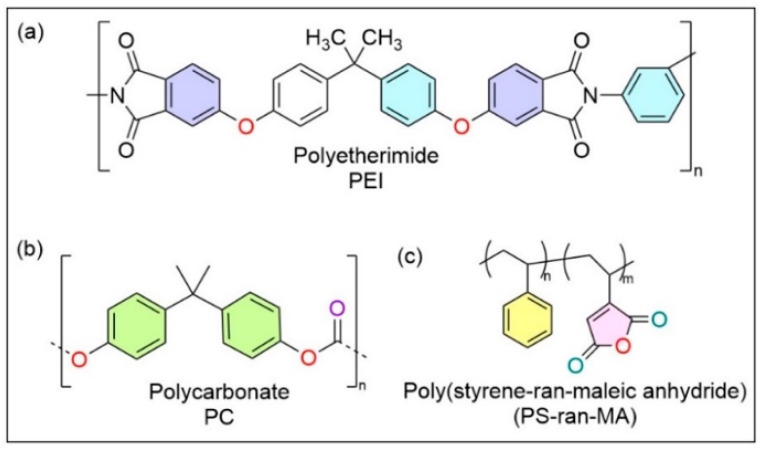
The chemical structure of (**a**) PEI, (**b**) PC, and compatibilizer shows the functional groups present in the structure that could be responsible for the interaction between the polymers and compatibilizer.

On comparing the spectra of neat PEI, PEI: PC (70:30), PEI: PC (70:30) X1, and ULTEM9085, it was observed that the imide ring confirmation of PEI is affected by the blending. The shift of the C=O peak in PEI: PC (70:30) blend is attributed to the favorable interaction of ether groups of PC (oxygen lone-pair electrons) with the electron-deficient imide rings in PEI. In the case of the PEI: PC (70:30) X1 blends, the interaction between the anhydride group of compatibilizer and imide group of PEI and or ether group of PC must have taken place. The representative chemical structure of the PEI, PC, and compatibilizer is shown in Figure 11.

## 4. Conclusions

High-performance polymers (HPPs), such as polyetherimide (PEI), hold significant promise for advanced 3D printing applications in aerospace, automotive, and medical industries due to their exceptional thermal and mechanical properties. However, widespread adoption is hindered by challenges in material compatibility and processability.

This study successfully developed PEI: PC blends with varying compositions (90:10, 70:30, 50:50, 10:90) using twin-screw extrusion for potential application as feedstock in fused filament fabrication (FFF). The incorporation of 1 wt% compatibilizer markedly enhanced the thermal stability and mechanical performance of the PEI: PC (70:30) blend while simultaneously reducing its viscosity, thereby improving processability. Employing the Taguchi method of design of experiments (DOE) to optimize the printing process, the study achieved printed parts with superior thermal and mechanical stability, particularly for the PEI: PC (70:30) X1 formulation.

The findings underscore the critical role of the compatibilizer in enhancing interfacial interactions between polymer phases, as evidenced by SEM and FTIR analysis. Notably, the developed filaments demonstrated properties comparable to, or even surpassing, those of commercially available ULTEM™9085. Considering the economic aspect, ULTEM™9085 filaments are priced at approximately 350–410 CAD per kilogram, while neat PEI pellets are available at a significantly lower cost of 25–35 CAD per kilogram.

These advancements highlight the potential of compatibilized PEI: PC blends as a cost-effective and high-performance alternative for 3D printing in demanding applications. Future research should focus on exploring varying compatibilizer contents and blend ratios to further elucidate their influence on material properties and expand the range of potential applications.

## Figures and Tables

**Table 1:** Sample codes based on the ratio of PEI: PC blends prepared by extrusion.

Code of Materials	PEI (wt%)	PC (wt%)	Compatibilizer (wt%)
Neat PEI	100	0	0
PEI: PC (90:10)	90	10	0
PEI: PC (70:30)	70	30	0
PEI: PC (50:50)	50	50	0
PEI: PC (10:90)	10	90	0
PEI: PC (70:30) X1	70	30	1
Neat PC	0	100	0

**Table 2:** Design of Experiments (DOE) used for the printing of the samples.

DOEs	Nozzle Temperature (°C)	Bed Temperature (°C)	Chamber Temperature (°C)
DOE1	340	180	100
DOE2	340	180	120
DOE3	340	180	130
DOE4	350	190	120
DOE5	350	190	130
DOE6	350	190	130
DOE7	360	200	100
DOE8	360	200	120
DOE9	360	200	130

**Table 3:** Glass transition (Tg) and melting temperature (Tm) of neat PEI and PEI: PC blends.

Code of Materials	Tg1 (°C)	Tg2 (°C)
	PC	PEI
Neat PEI	---	212.0
PEI: PC (90:10)	---	211.4
PEI: PC (70:30)	144.5	211.9
PEI: PC (50:50)	145.4	220.8
PEI: PC (10:90)	145.1	220.8
PEI: PC (70:30) X1	148.5	217.6
ULTEM9085	139.9	180.4

## Data Availability

The original contributions presented in the study are included in the article/Supplementary Material, further inquiries can be directed to the corresponding author.

## Supplementary Materials

The following supporting information can be downloaded at: https://www.mdpi.com/article/10.3390/polym16233384/s1, Table S1: Degradation behavior of PEI: PC blends under air and nitrogen and their comparison with commercial ULETEM9085. Figure S1: DSC thermograms of neat PEI, PEI: PC blends, PEI: PC (70:30) X1, and ULTEM9085. Figure S2: Storage modulus at different DOEs of PEI: PC (70:30) blends. Figure S3. (a) main effects plot for SN ratio and (b) main effects plot for means.
