# Supplementary material for: High-Performance Polymer Blends: Manufacturing of Polyetherimide (PEI)–Polycarbonate (PC)-Based Filaments for 3D Printing
Source: Polymers (Basel). 2024 Nov 30;16(23):3384. doi: 10.3390/polym16233384 (PMC11644298; doi:10.3390/polym16233384)
Supplement: Supplementary file 1 [file polymers-16-03384-s001.zip › polymers-3329405-supplementary.pdf]

polymers

MDPI

Article

High-Performance Polymer Blends: Manufacturing of Polyetherimide (PEI)–Polycarbonate (PC)-Based Filaments for 3D Printing

Shikha Singh [1,2,*] and Pascal Hubert [1,2]

1 Department of Mechanical Engineering, McGill University, Montreal, QC H3A 0C3, Canada; pascal.hubert@mcgill.ca
2 CREPEC—Research Centre for High-Performance Polymer and Composite Systems, Montreal, QC H3A 0C3, Canada
* Correspondence: shikha.singh@mcgill.ca; Tel.: +1-514-550-5632

Table S1. Degradation behaviour of PEI: PC blends under air and nitrogen and their comparison with commercial ULETEM9085.

Code of materials	Under Air		Under Nitrogen		Char yield (%)	Residue (%)	LOI (%)
	Td (5%)	Td (30%)	Td (5%)	Td (30%)			
Neat PEI	536	610	564	597	0.12	52	38.3
PEI: PC (90:10)	492	591	524	581	0.21	44	35.1
PEI: PC (70:30)	488	542	528	563	0.11	37	32.3
PEI: PC (50:50)	474	524	522	556	0.53	36	31.9
PEI: PC (10:90)	477	515	514	544	0.25	26	27.9
PEI: PC (70:30) X1	498	549	499	538	0.98	38	32.7

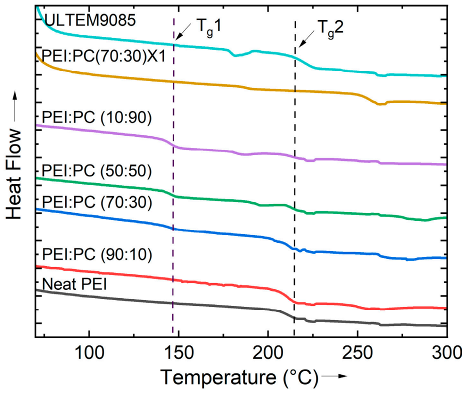

Figure S1. DSC thermograms of neat PEI, PEI: PC blends, PEI: PC (70:30) X1 and ULTEM9085.

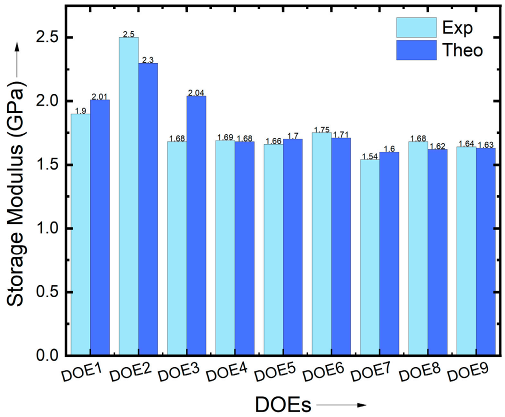

Figure S2. A comparison of experimental and theoretical storage modulus of PEI: PC (70:30) blends at different DOEs to understand the effect of nozzle temperature, bed temperature, and chamber temperature on the storage modulus.

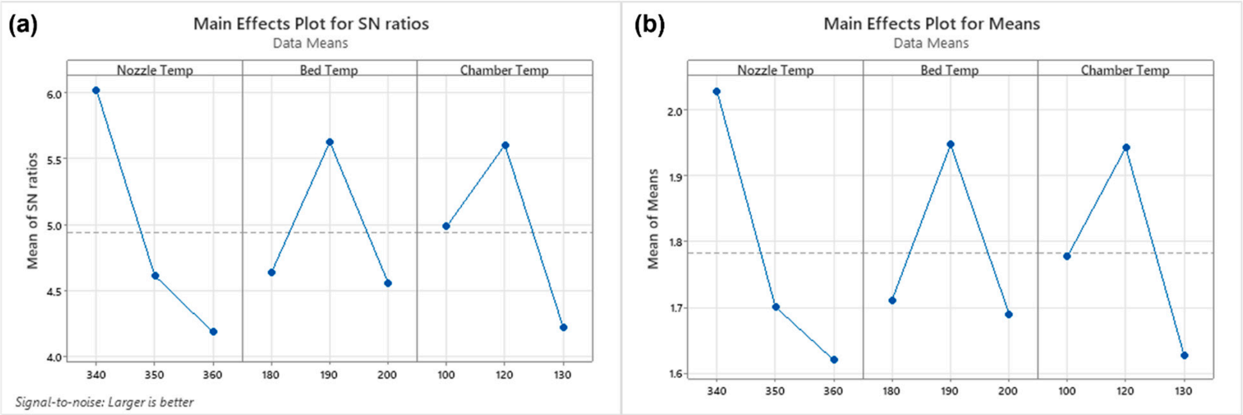

Figure S3. (a) Main effects plot for SN ratio and (b) main effects plot for means.
